# Determining whether the effect of liraglutide on non‐alcoholic fatty liver disease depends on reductions in the body mass index

**DOI:** 10.1002/jgh3.12384

**Published:** 2020-06-30

**Authors:** Megumi Shiomi, Yoichi Tanaka, Tesshu Takada, Katsuya Otori

**Affiliations:** ^1^ Department of Clinical Pharmacy, School of Pharmacy Kitasato University Minato‐ku Japan; ^2^ Department of Pharmacy Kitasato University Medical Center Saitama Japan; ^3^ Department of Endocrinology, Diabetes, and Metabolism, School of Medicine Kitasato University Sagamihara Japan

**Keywords:** body mass index, liraglutide, non‐alcoholic fatty liver disease, type 2 diabetes mellitus

## Abstract

**Background and Aim:**

Non‐alcoholic fatty liver disease (NAFLD) initially presents as steatosis, which can progress to non‐alcoholic steatohepatitis (NASH), and often presents clinically alongside metabolic syndromes. Glucagon‐like peptide‐1 receptor agonists (GLP‐1 RAs) are regularly utilized to treat type 2 diabetes mellitus. The GLP‐1 RA—liraglutide—ameliorates liver enzymes, histological features, and liver fat content of patients with NASH. However, few studies have examined whether the effect of GLP‐1 RAs depends on changes in the patient's body mass index (BMI). Therefore, this retrospective study aimed to investigate whether the efficacy of liraglutide depended on the baseline BMI or a reduction in BMI.

**Methods:**

Fifty‐five Japanese patients with type 2 diabetes mellitus and NAFLD who received liraglutide treatment for 24 weeks were assessed. The association between BMI and liver function or fibrosis was evaluated based on the aspartate aminotransferase, alanine aminotransferase, and fibrosis‐4 indices.

**Results:**

We found that 24 weeks of liraglutide treatment improved liver function and fibrosis in patients with type 2 diabetes mellitus and NAFLD, regardless of BMI changes or obesity status.

**Conclusions:**

Our findings provide important insight into the impact of BMI on liver function and fibrosis in patients with type 2 diabetes mellitus and NAFLD who are treated with liraglutide.

## Graphical abstract

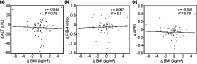

This study aimed to determine whether the effect of liraglutide in improving nonalcoholic fatty liver disease (NAFLD) depended on the baseline body mass index (BMI) or a reduction in BMI. Baseline BMI and Δ BMI were not independent predictors of changes in liver function and fibrosis index. This study highlighted that 24 weeks of liraglutide treatment improved liver function and liver fibrosis in patients with type 2 diabetes and NAFLD, regardless of BMI change and obesity status.

## Introduction

Glucagon‐like peptide‐1 receptor agonists (GLP‐1 RAs) enhance glycemic control via glucose concentration‐dependent insulin secretion and inhibition of glucagon secretion^1^ and promote weight loss by decreasing a patient's appetite.^2^ They are safe, effective, and widely used in clinics to treat type 2 diabetes mellitus (T2DM).^1^

Non‐alcoholic fatty liver disease (NAFLD), which initially develops as steatosis that progresses to non‐alcoholic steatohepatitis (NASH), is prevalent in 25.24% of individuals globally and in 27.37% of Asians.^3^ NAFLD often presents clinically alongside a metabolic syndrome.^3^ In particular, T2DM is an independent predictor of advanced fibrosis and overall liver‐related mortality.^4^, ^5^ The high mortality rate of NAFLD highlights the importance of early intervention. Weight loss improves liver function and, thus, is crucial in NAFLD treatment.^6^ Thiazolidinediones and vitamin E also appear beneficial.^7^ However, till date, no medication has been approved for the treatment of NAFLD. In the liraglutide efficacy and action in NASH (LEAN) study, the GLP‐1 RA liraglutide led to the histological resolution of NASH.^8^ Liraglutide ameliorates liver enzymes, histological features, and liver fat content.^9^, ^10^, ^11^ These effects may be due to improvements in weight loss, which is a consistent finding in GLP‐1 RA treatment.^2^ However, few studies have investigated whether the effect of GLP‐1 RAs depends on changes in a patient's body mass index (BMI). NAFLD and NASH have also been observed in individuals with normal BMIs,^12^ and treatments other than weight loss may be necessary. Thus, this study aimed to investigate the efficacy of liraglutide in improving NAFLD and to determine whether its effect depended on baseline BMI or a reduction in BMI. Accordingly, we evaluated the association between BMI and liver function or fibrosis using noninvasive scores in patients with T2DM and NAFLD who were treated with liraglutide.

## Methods

### *Subjects*

This retrospective study included 55 Japanese patients with NAFLD and T2DM who received liraglutide treatment at Kitasato University Medical Center (November 2010 to February 2018). NAFLD diagnostic criteria were: (i) evidence of fatty liver disease on ultrasonography; (ii) no more than 20 g alcohol/day for women or 30 g alcohol/day for men; (iii) negative for hepatitis B and C; and (iv) absence of autoimmune and drug‐induced liver diseases. We excluded patients (i) who received liraglutide treatment for <24 weeks; (ii) experienced the addition of, or change in, other hypoglycemic agents (HAs), antihypertensive agents, and antidyslipidemic agents from 8 weeks pre‐ to 24 weeks postliraglutide treatment; (iii) who received a higher dose of other HAs, antihypertensive agents, and antidyslipidemic agents from 8 weeks pre‐ to 24 weeks postliraglutide treatment; (iv) who received other GLP‐1 RAs before starting liraglutide; (v) had poor drug compliance based on regular prescription requests obtained from their medical records; (vi) with a history of taking agents that effect NAFLD (e.g., vitamin E, sodium‐glucose cotransporter 2 inhibitors, and thiazolidinediones);^7^, ^13^ and (vii) with mental health diagnoses or patients taking psychotropic agents.

### *Study design and liraglutide treatment*

This study was approved by the Ethical Committee of Kitasato University Medical Center (registration number 29–15) and was conducted following the principles of the Declaration of Helsinki. Written informed consent was obtained from all patients.

Patients were initially administered 0.3 mg liraglutide once daily for 1 or 2 weeks, followed by 0.6 mg once daily for 1 or 2 weeks, and finally 0.9 mg once daily. In Japan, 0.9 mg was the maximum approved dose for the duration of the study, and dose administration depended on the severity of gastrointestinal disorders, which are the primary adverse effects of the drug. The patients' diets were managed by a registered dietician throughout the treatment period.

### *Assessments*

The following pre‐ and post‐treatment (after 24 weeks) clinical data were obtained: diabetic complications (nephropathy and retinopathy); use of HAs; BMI; glycated hemoglobin (HbA1c) level; diastolic and systolic blood pressure; levels of high‐density and low‐density lipoprotein‐cholesterol (HDL‐C, LDL‐C); triglyceride (TG) levels; triglyceride to high‐density lipoprotein‐cholesterol ratio (TG/HDL‐C), an alternative indicator of insulin resistance;^14^ estimated glomerular filtration rate (eGFR); aspartate aminotransferase (AST), alanine aminotransferase (ALT), and γ‐glutamyl transferase (GGT) levels; and platelet counts. Diabetic retinopathy was defined as at least simple retinopathy. Diabetic nephropathy was defined as a urine albumin‐to‐creatinine ratio ≥30 mg/g creatinine and/or eGFR<30 mL/min/1.73 m^2^.^15^ BMI was assessed by kg/m^2^, and obesity was defined as BMI ≥25 kg/m^2^, based on the Japan Society for the Study of Obesity guidelines.^16^ The HbA1c values were recorded as the National Glycohemoglobin Standardization Program values,^17^ and eGFR was calculated using the Japan Nephrology Society equation.^18^ To assess the status of liver fibrosis, we used two noninvasive scores of liver fibrosis: the fibrosis‐4 (FIB‐4) index (formula: age [years] × AST [U/L])/(platelet count [10^9^/L] × (ALT [U/L]^1/2^))^19^ and the aspartate aminotransferase‐to‐platelet ratio index (APRI; formula: AST [U/L]/upper limit of AST (35 U/L)/platelet count [10^9^/L] × 100).^20^ To assess for baseline advanced fibrosis, the risks of advanced fibrosis were categorized as low (<1.3), intermediate (1.30–2.67), and high (>2.67) according to the FIB‐4 index^19^ and as low (<0.5) and high (>1.5) according to the APRI.^20^

### *Statistical analysis*

Data were expressed as the mean ± SD, median and interquartile range (IQR), or numbers and percentages. Changes in baseline parameters and at 24 weeks after treatment were compared using the Wilcoxon signed‐rank test or paired *t*‐test for nonnormally or normally distributed data, respectively. Changes in ALT (ΔALT), FIB‐4 index (ΔFIB‐4 index), APRI (ΔAPRI), and BMI (ΔBMI) were calculated as the week 24 value minus the baseline value. Parameters with skewed distributions were transformed into a natural logarithm before performing correlation and regression analyses. Pearson's correlation coefficient analysis was used to assess the relationships among ΔALT, ΔFIB‐4 index, ΔAPRI, and explanatory variables. Multiple regression analyses were performed to examine whether BMI was independently associated with ΔALT, ΔFIB‐4 index, and ΔAPRI. We included ΔALT, ΔFIB‐4 index, and ΔAPRI as dependent variables. Moreover, BMI and HbA1c, which are clinical outcome indicators of GLP‐1 RAs, and values with Pearson's correlation coefficients (*P*) <0.05 were included as explanatory variables. Multicollinearity of variables was checked using the variance inflation factor (VIF), with serious collinearity defined as VIF >10. A subgroup analysis was further performed based on the three categories of ΔBMI. One‐way analysis of variance and the Kruskal‐Wallis test were used to compare each variable among the three groups. All statistical analyses were conducted using the R software (version 3.4.1, the R Foundation for Statistical Computing, Vienne, Austria),^21^ and *P‐*values of <0.05 were considered significant.

## Results

### *Patients' characteristics*

A total of 55 patients with a mean age of 55.6 ± 12.9 years were included in this study. Table 1 presents the patients' baseline characteristics. The mean duration of T2DM was 11.4 ± 8.3 years. A total of 47 (85%) patients were obese; 34 (62%), 18 (33%), and 3 (5%) patients had a low, intermediate, and high risk of advanced fibrosis, respectively, according to the FIB‐4 index. APRI scores revealed 42 (76%) patients had low‐risk advanced fibrosis; no patients were classified as high risk. The median liraglutide dose after 24 weeks was 0.9 mg.

**Table 1:** Patients' baseline characteristics (*n* = 55)

Characteristics	Baseline
Male, *n* (%)	22 (40)
Age (years)	55.6 ± 12.9
Duration of diabetes (years)	11.4 ± 8.3
Obesity, *n* (%)	47 (85)
Diabetes complications, *n* (%)	
Retinopathy	19 (35)
Nephropathy	16 (29)
Previous antidiabetic treatment, *n* (%)	
Diet only	4 (7)
Sulfonylureas	32 (58)
Metformin	39 (71)
α‐Glycosidase inhibitors	16 (29)
DPP‐4 inhibitors	20 (36)
Insulin	13 (24)
Hypoglycemic agents concomitant with starting liraglutide, *n* (%)	
Sulfonylureas	31 (56)
Metformin	18 (33)
α‐Glycosidase inhibitors	5 (9)
Insulin	9 (16)
FIB‐4 index, *n* (%)	
<1.30	34 (62)
1.30–2.67	18 (33)
>2.67	3 (5)
APRI, *n* (%)	
<0.5	42 (76)
0.5–1.5	13 (24)
>1.5	0 (0)

Data are expressed as mean ± standard deviation or numbers and percentages. APRI, aspartate aminotransferase to platelet counts ratio index; DPP‐4, dipeptidyl peptidase‐4; FIB‐4 index, fibrosis‐4 index.

### *Post‐treatment changes*

**Figure 1:**
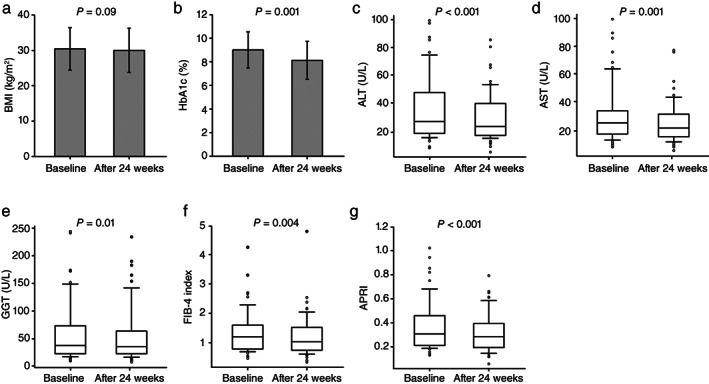
Changes in patients' characteristics from baseline to 24 weeks after liraglutide treatment. Distribution of body mass index (BMI) (a), glycated hemoglobin (HbA1c) (b), alanine aminotransferase (ALT) (c), aspartate aminotransferase (AST) (d), γ‐glutamyl transferase (GGT) (e), fibrosis‐4 (FIB‐4) index (f), and aspartate aminotransferase to platelet ratio index (APRI) (g) at baseline and after 24 weeks. Boxes and horizontal lines represent interquartile ranges (IQR) and median values, respectively. The upper and lower whiskers indicate the 10th and 90th percentiles, while open dots represent outliner values. (a) The mean BMI values at baseline and 24 weeks were 30.4 ± 6.0 and 29.9 ± 6.2, respectively. (b) The mean HbA1c values at baseline and 24 weeks were 9.0 ± 1.5 and 8.1 ± 1.6, respectively. (c) The median ALT values at baseline and 24 weeks were 27.0 (IQR: 18.5–48.0) and 23.0 (IQR: 17.0–37.0), respectively. (d) The median AST values at baseline and 24 weeks were 25.0 (IQR: 18.5–32.0) and 22.0 (IQR: 17.0–30.0), respectively. (e) The median GGT values at baseline and 24 weeks were 38.0 (IQR: 23.0–73.5) and 36.0 (IQR: 23.0–64.0), respectively. (f) The median FIB‐4 indices at baseline and 24 weeks were 1.20 (IQR: 0.80–1.60) and 1.03 (IQR: 0.75–1.51), respectively. (g) The median APRIs at baseline and 24 weeks were 0.31 (IQR: 0.21–0.45) and 0.28 (IQR: 0.20–0.39), respectively. The *P*‐value was determined using a paired *t*‐test or Wilcoxon signed‐rank test.

Table S1, Supporting information shows the changes in patient characteristics from baseline to 24 weeks post‐treatment. The mean HbA1c significantly decreased after 24 weeks (*P* = 0.001). Furthermore, both liver function parameters [median ALT (*P* < 0.001), AST (*P* = 0.001), and GGT (*P* = 0.01)] and liver fibrosis [median FIB‐4 index (*P* = 0.004) and APRI (*P* < 0.001)] significantly decreased after 24 weeks (Fig. 1). Meanwhile, the mean BMI decreased from 30.4 ± 6.0 kg/m^2^ to 29.9 ± 6.2 kg/m^2^, but this was not statistically significant.

### *Correlation between liver function or fibrosis index and clinical characteristics*

**Figure 2:**
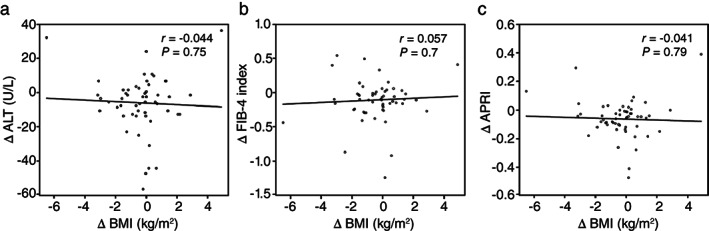
Correlation between liver function or fibrosis index and clinical characteristics. Correlation between change in alanine aminotransferase (ΔALT) (a), fibrosis‐4 index (ΔFIB‐4 index) (b), or aspartate aminotransferase to platelet ratio index (ΔAPRI) (c) and body mass index (ΔBMI). *r*, the correlation coefficient was identified using Pearson's correlation coefficient analysis.

Table S2 shows the correlation between ΔALT, ΔFIB‐4 index, and ΔAPRI and baseline characteristics. For the ΔFIB‐4 index and ΔAPRI, we excluded the parameters included in the formula as explanatory variables. However, considering the confounding factor, we added the baseline ALT, baseline FIB‐4 index, and baseline APRI to the explanatory variables for correlation analysis with ΔALT, ΔFIB‐4 index, and ΔAPRI, respectively. ΔALT was significantly positively correlated with age (*r* = 0.292, *P* = 0.03) but negatively correlated with BMI (*r* = −0.295, *P* = 0.03), Log ALT (*r* = −0.642, *P* < 0.001), and Log AST (*r* = −0.570, *P* < 0.001). ΔFIB‐4 index was significantly positively correlated with HDL‐C (*r* = 0.329, *P* = 0.02) but negatively correlated with the Log FIB‐4 index (*r* = −0.323, *P* = 0.03). ΔAPRI was significantly and negatively correlated with Log ALT (*r* = −0.572, *P* < 0.001) and Log APRI (*r* = −0.621, *P* < 0.001). Meanwhile, ΔAPRI, ΔFIB‐4, and ΔALT indices were not significantly correlated with obesity. No significant correlation with ΔALT, ΔFIB‐4 index, or ΔAPRI and ΔBMI was revealed (Fig. 2). ΔAPRI, ΔFIB‐4, and ΔALT did not show a significant correlation with changes in other parameters 24 weeks after treatment.

### *Multiple regression analysis of factors associated with liver function and fibrosis index*

To examine whether BMI is independently associated with ΔALT, ΔFIB‐4 index, and ΔAPRI, a multiple regression analysis was performed using BMI, HbA1c, and variables with *P* < 0.05 in Pearson's correlation coefficient analysis as explanatory variables. All models had VIF values <10. Multiple regression analyses showed that ALT and FIB‐4 index baselines were independent factors of ΔALT (*β*, −43.112; *P* = 0.009) and ΔFIB‐4 index (*β*, −0.531; *P* = 0.01), respectively, while ALT and APRI baselines were independent factors of ΔAPRI (*β*, −0.196; *P* = 0.03; *β*, −0.262; *P* = 0.007) (Table 2). Meanwhile, baseline BMI was not an independent factor of ΔALT, ΔFIB‐4 index, and ΔAPRI (Table 2). Parameters exhibiting changes at 24 weeks did not show significance in Pearson's correlation coefficient analysis; thus, a multiple regression analysis was not performed.

**Table 2:** Influencing factors of ALT, FIB‐4 index, and APRI as identified via multiple regression analysis

Variable	*β*	*t*‐value	*P*‐value
ΔALT	Adjusted *R* ^2^ = 0.393, *P* < 0.001		
Age (years)	−0.065	−0.368	0.71
BMI (kg/m^2^)	−0.235	−0.696	0.49
HbA1c (%)	0.806	0.639	0.53
Log ALT (U/L)	−43.112	−2.689	0.009
Log AST (U/L)	−2.421	−0.143	0.89
ΔFIB‐4 index	Adjusted *R* ^2^ = 0.110, *P* = 0.06		
BMI (kg/m^2^)	−0.009	−1.184	0.24
HbA1c (%)	−0.002	−0.06	0.95
HDL‐C (mmol/L)	0.006	1.437	0.16
Log FIB‐4 index	−0.531	−2.485	0.01
ΔAPRI	Adjusted *R* ^2^ = 0.452, *P <* 0.001		
BMI (kg/m^2^)	−0.002	−0.667	0.51
HbA1c (%)	0.004	0.344	0.73
Log ALT (U/L)	−0.196	−2.201	0.03
Log APRI	−0.262	−2.831	0.007

*β*, standard regression coefficient; ALT, alanine aminotransferase; APRI, aspartate aminotransferase to platelet counts ratio index; AST, aspartate aminotransferase; BMI, body mass index; FIB‐4 index, fibrosis‐4 index; HbA1c, glycated hemoglobin; HDL‐C, high‐density lipoprotein cholesterol; *R* ^2^, multiple correlation coefficient.

### *Changes in liver function and fibrosis index in the three groups according to change in* *BMI*

Variations in liver function and fibrosis index 24 weeks after liraglutide administration were evaluated among the three patient cohorts, grouped based on weight change tertiles (Table 3). To examine the association of ΔBMI with ΔALT, ΔFIB‐4 index, and ΔAPRI, we performed a subgroup analysis and divided the patients into three groups according to ΔBMI (ΔBMI ≤−0.67 kg/m^2^, >−0.67 kg/m^2^ to ≤0.11 kg/m^2^, and >0.11 kg/m^2^). There was no statistical difference in baseline BMI, baseline ALT, baseline FIB‐4 index, and baseline APRI among the three groups (Table 3). Meanwhile, ΔBMI significantly differed among the three groups (*P* < 0.001). ΔALT, ΔFIB‐4 index, and ΔAPRI decreased in each group, although not significantly (Table 3). Therefore, there was no association between changes in liver function and fibrosis and changes in BMI.

**Table 3:** Changes in ALT, FIB‐4 index, and APRI in the three BMI groups

Variable	ΔBMI (kg/m^2^)			*P*‐value
	ΔBMI ≤ −0.67	−0.67 < ΔBMI ≤0.11	0.11 < ΔBMI	
	(*n* = 18)	(*n* = 19)	(*n* = 18)	
BMI (kg/m^2^)				
Baseline BMI	29.8 ± 6.5	31.0 ± 6.6	30.3 ± 5.0	0.84
ΔBMI	−2.2 ± 1.4	−0.2 ± 0.2	1.2 ± 1.2	<0.001
ALT (U/L)				
Baseline ALT	27.0 (17.0–35.8)	25.0 (20.0–62.0)	31.0 (21.3–66.8)	0.36
ΔALT	−4.9 ± 11.3	−9.3 ± 18.4	−7.8 ± 19.3	0.73
FIB‐4 index				
Baseline FIB‐4 index	1.51 (1.15–2.14)	1.12 (0.95–1.21)	0.99 (0.74–1.60)	0.1
ΔFIB‐4 index	−0.07 ± 0.35	−0.14 ± 0.27	−0.17 ± 0.39	0.68
APRI				
Baseline APRI	0.37 (0.23–0.44)	0.27 (0.21–0.43)	0.32 (0.21–0.49)	0.88
ΔAPRI	−0.03 ± 0.11	−0.09 ± 0.08	−0.08 ± 0.2	0.41

Data are expressed as mean ± standard deviation or median and interquartile range. Statistical significance was estimated using a one‐way analysis of variance or Kruskal‐Wallis test.

ALT, alanine aminotransferase; APRI, aspartate aminotransferase to platelet counts ratio index; BMI, body mass index; FIB‐4 index, fibrosis‐4 index.

## Discussion

This study showed that a 24‐week liraglutide treatment improved liver function and fibrosis independent of baseline BMI and BMI changes in patients with T2DM and NAFLD. The effects of treatment were associated with baseline liver function and the noninvasive liver function scores. Although the mean BMI decreased at 24 weeks after liraglutide treatment, this did not affect liver function and fibrosis. Similar findings were observed in the subgroup analysis according to BMI change. To the best of our knowledge, this is the first study to clarify that baseline BMI and its subsequent changes do not influence the effects of liraglutide treatment in patients with NAFLD. These findings complement those of previous studies, which showed that liraglutide improved the ALT, AST, APRI, and histological features of patients with type 2 diabetes and NAFLD.^8^, ^9^, ^10^, ^11^

Although liver biopsy is the gold standard for diagnosing NAFLD/NASH, this procedure is difficult to perform in these patients due to its highly invasive nature; moreover, it increases the risk for sampling errors and postprocedure complications.^22^ Ultrasonography is used more often for screening fat deposition because it is relatively inexpensive and safe. Recently, several simple, noninvasive scoring systems—including commonly recorded laboratory and clinical assessments—have been developed and validated for the assessment of advanced liver fibrosis in patients with NAFLD.^22^, ^23^ In Japanese patients, the FIB‐4 index showed superior accuracy to other scoring systems for diagnosing progressive fibrosis in patients with NAFLD, followed by the NAFLD fibrosis score (NFS) and APRI.^24^ Here, we applied the FIB‐4 index and APRI to assess for liver fibrosis. Although NFS is also effective for detecting advanced fibrosis, we could not calculate the NFS because serum albumin—one of its components^25^—was available only in a few patients. GLP‐1 RAs are associated with an improvement in liver fibrosis,^9^ and our study demonstrates a decrease in both FIB‐4 index and APRI. Therefore, a low‐dose liraglutide treatment (0.9 mg) may improve AST, ALT, and liver fibrosis.

Weight loss is the primary recommended treatment for NAFLD, and weight loss of at least 7% can lead to significant ALT and histological improvements.^6^ The pathways through which GLP‐1 RAs improve NAFLD are not completely known, but it has an indirect effect of weight loss due to the association between weight loss and increased insulin sensitivity, decreased circulating free fatty acid, and improved chronic inflammatory status.^26^, ^27^, ^28^ Furthermore, it exhibits a direct effect on the lipid metabolism of hepatocytes; enhances β oxidation of fatty acids; and improves oxidative stress, endoplasmic reticulum stress, inflammatory stress, and insulin sensitivity.^26^, ^27^, ^28^, ^29^ Here, there was no significant decrease in BMI 24 weeks after liraglutide treatment, and no significant association was demonstrated between liver function or the fibrosis index and weight loss. A post‐hoc analysis of the LEAN study analyzed 23 patients who were treated with 1.8 mg of liraglutide. We found no difference in weight changes between those with and without histological improvements.^8^ A LEAN substudy demonstrated that treatment with liraglutide (1.8 mg) decreased hepatic de novo lipogenesis, an important contributor to the accumulation of hepatic fat in NAFLD, independent of weight loss.^30^ In addition, Cuthbertson *et al*. ^31^ reported that GLP‐1 RAs, including liraglutide (1.2 mg), were not significantly correlated with changes in body weight and intrahepatic lipids. However, these studies included only a small number of patients with T2DM who were treated with liraglutide. In a larger prospective study that included patients with uncontrolled T2DM, treatment with liraglutide (1.2 mg) significantly reduced liver fat content, which was mainly driven by body weight loss.^10^

Liraglutide induces weight loss in a dose‐dependent manner, and the maximum‐approved dose for the treatment of obesity is 3 mg.^32^ A recent study that investigated the effects of liraglutide on the BMI of patients with T2DM reported weight loss and amelioration of fatty liver disease. However, these patients did not present with concomitant NAFLD, and patients undergoing synchronous treatments that could have interfered with the results were not excluded prior to the study.^33^ Another recent study revealed that a higher dose of liraglutide (3 mg daily over 26 weeks) was as effective as exercise and dieting for reducing insulin resistance, hepatic steatosis, and hepatocyte damage in obese patients with NAFLD. Notably, this cohort did not include patients with both NAFLD and T2DM.^34^ The discrepancies in the findings of these reports and those of this study may be due to differences in the number of patients and baseline characteristics such as age and gender. We did not evaluate intrahepatic fat content, but the liraglutide dose used in our study was smaller than that in previous studies. Hence, our findings indicate that NAFLD improvement after treatment with liraglutide (0.9 mg) may be due to a direct hepatic effect. Further studies are warranted to elucidate the exact mechanism of this hypothesized effect. Furthermore, the effects of liraglutide were independent of baseline BMI and obesity in our study. Therefore, liraglutide may be effective in patients with NAFLD, regardless of BMI status.

Insulin resistance is an important predisposing factor and is believed to influence the development of NAFLD.^26^, ^27^ A hyperinsulinemic–euglycemic clamp is the gold standard for evaluating insulin resistance, but the homeostasis model assessment of insulin resistance (HOMA‐IR) is convenient and, therefore, more widely used.^35^ In this study, we used TG/HDL‐C as a more reasonable surrogate of insulin resistance, and no significant association was found between the improvement of liver function and fibrosis index and insulin resistance. The positive effect of liraglutide (0.9 mg) on NAFLD may be attributed to the direct hepatic effect on hepatocyte lipid metabolism not mediated by insulin sensitization. However, liraglutide (1.8 mg) enhanced hepatic insulin sensitivity independent of weight loss.^30^ Hence, further studies using clamp test or HOMA‐IR are required to examine the association between liraglutide and insulin resistance.

This study has several limitations. First, although fatty liver disease and liver fibrosis were assessed via ultrasonography and a noninvasive scoring index, respectively, it is difficult to accurately determine the degree of fibrosis. Recently, transient elastography has been considered useful for noninvasive measurement and staging of liver fibrosis.^36^ Considering the result of the present study, adding transient elastography for the evaluation would be beneficial for accurately investigating the association between liver fibrosis and BMI in patients with T2DM and NAFLD who are treated with liraglutide. Second, our study period was 24 weeks, which may have been inadequate to observe the long‐term effects of liraglutide on NAFLD. However, after 24 weeks of treatment, many patients received additional medications or modified types and doses of HAs, antihypertensive agents, and antidyslipidemic agents. Hence, it was difficult to accurately assess the relationship between improvement of NAFLD and BMI. Accordingly, we could not perform a long‐term evaluation. Finally, we used a small study sample. Consequently, there were only a few patients with severe fibrosis who had a high FIB‐4 index and APRI. Therefore, a study with a larger patient sample size is required to confirm our results.

## Conclusion

This study highlighted that 24 weeks of liraglutide treatment improved liver function and liver fibrosis in patients with type 2 diabetes and NAFLD, regardless of BMI change and obesity status. Our findings provide important insight into the influence of BMI on liver function and fibrosis in patients with type 2 diabetes and NAFLD treated with liraglutide and help to understand the clinical effects of liraglutide on such patients.

## Declaration of conflict of interest

All authors declare that they have no potential conflicts including financial interests, activities, relationships, and affiliations.

## Author contributions

Megumi Shiomi initiated and designed the study, interpreted findings, and drafted the manuscript. Yoichi Tanaka designed the study and conducted statistical analysis. Tesshu Takada designed the study and reviewed the manuscript. Katsuya Otori designed the study and reviewed the manuscript. All authors participated in the writing of the manuscript and approved the final version of the manuscript.

## Supporting information


**Table S1.** Changes in clinical parameters from baseline to after 24 weeks of liraglutide treatment.

**Table S2.** Pearson's correlation coefficient analysis of the association between changes in ALT, FIB‐4 index, or APRI and baseline clinical characteristics.
