# Supplementary material for: Determining whether the effect of liraglutide on non‐alcoholic fatty liver disease depends on reductions in the body mass index
Source: JGH Open. 2020 Jun 30;4(5):995–1001. doi: 10.1002/jgh3.12384 (PMC7578289; doi:10.1002/jgh3.12384)
Supplement: Supplementary file 2 — Table S2. Pearson's correlation coefficient analysis of the association between changes in ALT, FIB‐4 index, or APRI and baseline clinical characteristics. [file JGH3-4-995-s002.docx]

**Supplementary Table 2. Pearson’s correlation coefficient analysis of the association between changes in ALT, FIB-4 index, or APRI and baseline clinical characteristics**

| Variable | Δ ALT | |  | Δ FIB-4 index | |  | Δ APRI | |
| --- | --- | --- | --- | --- | --- | --- | --- | --- |
|  | *r* | *P*-value |  | *r* | *P-*value |  | *r* | *P-*value |
| Sex, male | 0.079 | 0.6 |  | 0.097 | 0.51 |  | 0.017 | 0.91 |
| Age (years) | 0.292 | 0.03 |  |  |  |  | 0.228 | 0.12 |
| Duration of diabetes (years) | 0.153 | 0.27 |  | −0.184 | 0.21 |  | 0.066 | 0.66 |
| Obesity | −0.112 | 0.42 |  | −0.195 | 0.19 |  | −0.114 | 0.45 |
| Diabetic retinopathy | 0.216 | 0.12 |  | −0.063 | 0.67 |  | 0.136 | 0.36 |
| Diabetic nephropathy | −0.121 | 0.45 |  | −0.149 | 0.37 |  | −0.013 | 0.94 |
| BMI (kg/m^2^) | −0.295 | 0.03 |  | −0.103 | 0.49 |  | −0.274 | 0.06 |
| HbA1c (%) | −0.169 | 0.22 |  | −0.052 | 0.73 |  | −0.157 | 0.29 |
| SBP (mmHg) | −0.008 | 0.96 |  | 0.261 | 0.09 |  | 0.075 | 0.63 |
| DBP (mmHg) | −0.190 | 0.18 |  | 0.128 | 0.40 |  | −0.099 | 0.51 |
| HDL-C (mmol/L) | 0.114 | 0.41 |  | 0.329 | 0.02 |  | 0.201 | 0.17 |
| LDL-C (mmol/L) | −0.107 | 0.44 |  | 0.125 | 0.40 |  | 0.067 | 0.65 |
| Log TG (mmol/L) | −0.023 | 0.84 |  | −0.221 | 0.15 |  | −0.236 | 0.11 |
| Log TG/HDL-C | −0.049 | 0.72 |  | −0.279 | 0.06 |  | −0.248 | 0.09 |
| eGFR (mL/min/1.73 m^2^) | −0.116 | 0.41 |  | 0.268 | 0.07 |  | −0.026 | 0.86 |
| Log ALT (U/L) | −0.642 | < 0.001 |  |  |  |  | −0.572 | < 0.001 |
| Log AST (U/L) | −0.570 | < 0.001 |  |  |  |  |  |  |
| Log GGT (U/L) | −0.257 | 0.08 |  | −0.104 | 0.52 |  | −0.298 | 0.06 |
| Platelet count (×10^3^/μL) | −0.242 | 0.10 |  |  |  |  |  |  |
| Log FIB-4 index |  |  |  | −0.323 | 0.03 |  |  |  |
| Log APRI |  |  |  |  |  |  | −0.621 | <0.001 |

*r*, correlation coefficient; ALT, alanine aminotransferase; APRI, aspartate aminotransferase to platelet counts ratio index; AST, aspartate aminotransferase; BMI, body mass index; DBP, diastolic blood pressure; eGFR, estimated glomerular filtration rate; FIB-4 index, fibrosis-4 index; GGT, γ-glutamyl transferase; SBP, systolic blood pressure; LDL, low-density lipoprotein cholesterol; HDL-C, high-density lipoprotein cholesterol; HbA1c, glycated hemoglobin; TG, triglyceride; TG/HDL-C, triglyceride to high-density lipoprotein–cholesterol ratio
